# Potential Effects of Prepubertal Exposure to Perfluorooctane Sulfonic Acid on the First Wave of Folliculogenesis in Young CD‐1 Mice

**DOI:** 10.1002/bdr2.70052

**Published:** 2026-05-06

**Authors:** Bounleut Phanavanh, Jalina Moore, Amy Inselman, Xiaoqing Li, Kyung Sung, Pei‐Hsuan Hung, Li You, Noriko Nakamura

**Affiliations:** ^1^ National Center for Toxicological Research, U.S. Food and Drug Administration Jefferson Arkansas USA; ^2^ Center for Biologics Evaluation and Research, U.S. Food and Drug Administration Silver Spring Maryland USA; ^3^ Center for Devices and Radiological Health, U.S. Food and Drug Administration Silver Spring Maryland USA; ^4^ Center for Veterinary Medicine, U.S. Food and Drug Administration Rockville Maryland USA

**Keywords:** estradiol levels, folliculogenesis, in utero exposure, mice, ovary, perfluorooctane sulfonic acid, prepubertal exposure

## Abstract

**Background:**

Perfluorooctane sulfonic acid (PFOS) belongs to a group of synthetic chemicals referred to as per‐ and polyfluoroalkyl substances (PFAS) that are known to degrade slowly. Exposure to PFOS has been reported to cause birth defects and disrupt female sex hormone levels in mice. However, to date, the direct effects of PFOS exposure on folliculogenesis in young animals have not been evaluated. Therefore, this study was conducted to examine the potential effects of in utero and prepubertal exposure to PFOS on follicle development in juvenile, female mouse pups.

**Methods:**

ICR dams and young, female mouse pups were dosed with 0.1 mg/kg PFOS or saline (vehicle) via gavage beginning on gestation day (GD) 6 (plug positive = GD 0) through GD 17 (*n* = 11–17 per group) [in utero exposure] or postnatal day (PND) 7 (PND 0 = day of birth) through PND 21 (*n* = 22–29 per group) [prepubertal exposure]. Ovaries and blood were then collected from a pup/litter on PND 28 for analysis.

**Results:**

There were no adverse effects of PFOS exposure observed in the measured endpoints upon in utero exposure; however, statistically significant differences following prepubertal exposure were noted for serum estradiol levels, the total number of primordial follicles, and the transcript levels of *Foxo1* and *Gdf9*.

**Conclusion:**

The preliminary findings suggest that prepubertal exposure to PFOS may affect follicle development by disrupting estradiol production and altering *Foxo1* and *Gdf9* gene expression. Further studies will be important to examine the mechanisms underlying prepubertal exposure to PFOS on folliculogenesis.

**Abbreviation**
PFOS: Perfluorooctane sulfonic acid

## 1 Introduction

Folliculogenesis occurs in the ovary and is the process by which oocytes mature into eggs capable of being fertilized (Hannon and Curry 2018). Follicles are composed of an immature oocyte surrounded by somatic granulosa and theca cells which provide crucial support and hormone production for the maturing oocyte. Ovarian follicle development begins from primordial follicles that progress to primary, secondary, antral, and finally to mature preovulatory follicles from which maturing oocytes are released during ovulation (Hernández‐Ochoa et al. 2018). Many factors, including drug/chemical exposures and hormone imbalances, are known to impair follicle development leading to infertility or difficulty in conceiving naturally (Hannon and Curry 2018).

Perfluorooctane sulfonic acid (PFOS) belongs to a group of synthetic chemicals known as per‐ and polyfluoroalkyl substances (PFAS) (EPA, https://www.epa.gov/wqc/aquatic‐life‐criteria‐perfluorooctane‐sulfonate‐pfos#:~:text=Perfluorooctane%20Sulfonate%20). PFAS are ubiquitous and can be found in the environment, as well as personal care products. They are known to degrade slowly and therefore, their accumulation in the body is a primary concern of PFAS exposure. Several studies have examined the effects of exposure to various PFAS compounds on aspects of pregnancy and reproductive development in adult mice. Exposure to PFOS has previously been reported to cause birth defects in pregnancy and disrupt female sex hormone levels in mice (Ou et al. 2021; Shi et al. 2024). Perfluorooctanoic acid (PFOA), another compound in the PFAS family, has also been reported to affect folliculogenesis in vivo and in vitro (Liu et al. 2024; Yang et al. 2022; Zhang et al. 2023). The PFOS exposure (0.1 mg/kg) on folliculogenesis has been examined in adult mice (Feng et al. 2015). However, no studies have investigated the effects of in utero or prepubertal exposure to PFOS on folliculogenesis in young mice. Developing mice have different drug sensitivity and responses compared to the adult mice (Narciso et al. 2017). Therefore, we conducted this study to examine the potential effects of PFOS exposure on the first wave of folliculogenesis using the mice dosed prenatally and prepubertally by investigating histology, estradiol levels, and transcript expression of follicle related markers.

## 2 Materials and Methods

### 2.1 Materials

Unless otherwise stated, all reagents were purchased from Fisher Scientific (Pittsburgh, PA, USA) and Millipore‐Sigma (St. Louis, MO, USA).

### 2.2 Animals and Treatments

#### 2.2.1 Prepubertal Exposure

**FIGURE 1:**
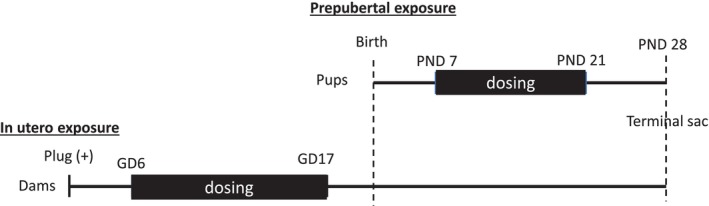
Experimental design of in utero and prepubertal exposure to PFOS.

Ten time‐mated ICR female mice (Hsd:ICR [CD‐1]; 8–9 weeks old) were purchased from Envigo (Indianapolis, IN, USA) and delivered to the National Center for Toxicological Research (NCTR) on gestation day (GD) 12. Male pups were culled, and litter sizes standardized at six to seven females. PFOS (cat. no.: 6164‐3‐08; Synquest Laboratories Inc., Alahcua, FL, USA) was used as the test compound. Female pups were administered by gavage a single daily dose of 0.1 mg/kg body weight PFOS in 0.9% saline from PND 7 through 21 as previously described (Feng et al. 2015) (*n* = 29). Mice in the control (CTRL) group were dosed with 0.9% saline (cat. no: 14208186; Aspen, Loveland, CO, USA) via gavage over the same time period (*n* = 22). Animals were weaned on PND 21 through 24; blood and ovaries were collected from one female pup per litter randomly at sacrifice on PND 28 (Figure 1).

#### 2.2.2 In Utero Exposure

Eighty female (7–8 weeks old) and 40 male (8–9 weeks old) ICR mice, per test compound, were purchased from Envigo to produce pregnant animals. After acclimation, one male for every two females was housed together for breeding. Plug positive dams were then housed two per cage until GD 15 when visibly identifiable pregnant dams were separated and housed individually. Plug‐positive dams were dosed with 0.1 mg/kg PFOS via gavage daily beginning on GD 6 (GD 0 = plug positive) and continuing through GD 17 (Feng et al. 2015) (*n* = 11). Mice in the control group were dosed with 0.9% saline via gavage over the same time period (*n* = 17). After birth, male pups were culled, and litter sizes were standardized to two females per litter. Blood and ovaries were collected from one female pup per litter randomly on PND 28 (Figure 1).

The animals were maintained under a 12:12 h light–dark cycle in a temperature (23°C ± 3°C) and humidity (50% ± 20%) controlled room. The dams were fed an NIH‐07 and/or NTP2000 diet, and water was provided *ad libitum*.

All animal procedures were approved by the NCTR Institutional Animal Care and Use Committee and followed the guidelines set forth by the National Research Council (2011) in the *Guide for the Care and Use of Laboratory Animals*.

### 2.3 Sample Collection

On PND 28, animals were euthanized by administering an excess of carbon dioxide followed by exsanguination via cardiac puncture. Blood was collected into ethylenediaminetetraacetic acid‐treated collection tubes (BD, Franklin Lakes, NJ, USA) and serum was obtained by centrifugation at 3000 *g* for 10 min. at room temperature. The serum was then stored at −80°C until use for estradiol measurements. Body weights and ovarian weights were obtained on PND 28. Ovaries were then fixed (see below) and used for follicle counting or were processed for qPCR analysis.

### 2.4 Follicle Counting

Ovaries were fixed in a 4% paraformaldehyde/phosphate buffer for 1–3 days at 4°C. Following fixation, 5 μm‐serial sections of paraffin‐embedded tissues were prepared according to standard histology procedures. The sections were mounted onto glass slides, deparaffinized in xylene, rehydrated in decreasing ethanol concentrations, stained with hematoxylin and eosin (H&E), coverslipped with Permount Mounting Medium (Thermo Fisher Scientific, Waltham, MA, USA), and examined under a light microscope. Every fifth section was counted from the first section containing follicular cells until the last section without follicular cells was observed. Only follicular cells that contained an oocyte with a nucleus were counted (prepubertal exposure: *n* = 9/control group, *n* = 12/PFOS group; in utero exposure: *n* = 11/control group, *n* = 8/PFOS group). Follicular cells were classified according to Pedersen and Peters (1968). Types 1 and 2 (primordial), 3a and 3b (primary), 4 (secondary), 5a and 5b (tertiary), and 6 (antral) were selected as the classifications.

### 2.5 Measurement of Serum Estradiol Levels

Serum estradiol levels were measured using an Estradiol ELISA kit per the manufacturer's protocol (cat. # ES380S; Calbiotech, El Cajon, CA, USA) (prepubertal exposure: *n* = 22/control group, *n* = 29/PFOS group; in utero exposure: *n* = 17/control group, *n* = 11/PFOS group).

### 2.6 RNA Extraction, cDNA Synthesis, and qPCR

RNA was extracted from whole ovaries using the miRNeasy Kit (Qiagen, Germantown, MD, USA) (prepubertal exposure: *n* = 22/control group, *n* = 29/PFOS group; in utero exposure: *n* = 17/control group, *n* = 11/PFOS group). After extraction, the concentration of each RNA sample was determined using a DS‐11 spectrophotometer (DeNovix Inc., Wilmington, DE, USA). Complementary DNA (cDNA) was synthesized using a SuperScript IV VILO Master Mix (cat. #. 11,766,050; Thermo Fisher Scientific, Carlsbad, CA, USA) using 1 μg of RNA per reaction. Quantitative polymerase chain reaction (qPCR) analysis was performed using a ViiA 7 Real‐Time PCR System (Applied Biosystems, Foster City, CA, USA) with PowerUp SYBR Green Master Mix (cat. #. A25742; Thermo Fisher Scientific) according to the manufacturer's instructions. The relative steady‐state transcript levels were calculated using threshold cycle (Ct) values with the following equation: relative quantity = 2^−ΔΔCt^ (Livak and Schmittgen 2001). Transcript levels were normalized to the β‐actin (*Actb*) gene as an endogenous control for each sample. The relative ratios of transcript levels were calculated for each sample, setting the values for the control group as one. The specific primer pairs used in this study are shown in Table 1 (Xiao et al. 2017; Frost et al. 2021). Genes examined included: oocyte marker: (DEAD‐box helicase 4 [*Ddx4*]); granulosa cell marker: (GATA binding protein 4 [*Gata4*]); follicle development markers: (forkhead box O1 [*Foxo1*]), (forkhead box O3 [*Foxo3*]), and (growth differentiation factor 9 [*Gdf9*]); steroidogenic enzyme marker: (cytochrome P450 family 11 subfamily A member 1 [*Cyp11a1*]); and apoptosis marker: (caspase 3 [*Casp3*]).

**TABLE 1:** Primer pairs for qPCR validation.

Genes	Primer sequences	Amplified size (bp)	GenBank accession
*Gdf9*	For: 5′‐ATG GCA CTT CCC AGC AAC TTC‐3′ Rev.: 5′‐TGC CTC AGA CTC CAC ATT TTC A‐3′	132	XM_030245570.2
*Casp3*	For: 5′‐GAG CTT GGA ACG GTA CGC TA‐3′ Rev: 5′‐GAG TCC ACT GAC TTG CTC CC‐3′	118	NM_001284409.1
*Foxo1*	For: 5′‐CGT CCT CGA ACC AGC TCA AA‐3′ Rev: 5′‐TAC ACC AGG GAA TGC ACG TC‐3′	104	NM_019739.3
*Foxo3*	For: 5′‐TGA AGG GAA GGA GCC GAG GTA‐3′ Rev: 5′‐GCT CTC TCC TCT CGA GCC CA‐3′	93	NM_001376967.1
*Ddx4*	For: 5′‐GCC GTG GAG GAT TTG GTC TA‐3′ Rev: 5′‐GGT AAG TGT CAC CAT TGC CTG‐3′	129	NM_001145885.1
*Gata4*	For: 5′‐GTT TTC TGG GAA ACT GGA GCT GG‐3′ Rev: 5′‐TGC TTT CTG CCT GCT ACA CAC‐3′	130	NM_001310610.1
*Actb*	For: 5′‐GAT CAG CAA GCA GGA GTA CGA‐3′ Rev: 5′‐AAA ACG CAG CTC AGT AAC AGT C‐3	86	NM_007393.5

### 2.7 Statistical Analysis

Statistical analysis was conducted based on the number of litters (one female pup per litter) per treatment group in this study. Pups' body weights at PNDs 1–21 showed average body weights per litter. Body and organ weights, follicle size, and serum estradiol levels are presented as the mean ± standard deviation (SD). Statistical analyses were performed using GraphPad Prism software (version 10; San Diego, CA, USA). The qPCR data were analyzed using a *t*‐test with Bonferroni correction (Hochberg 1988; Shaffer 1995). A *p*‐value of < 0.05 was considered statistically significant.

## 3 Results

### 3.1 In Utero Exposure

**FIGURE 2:**
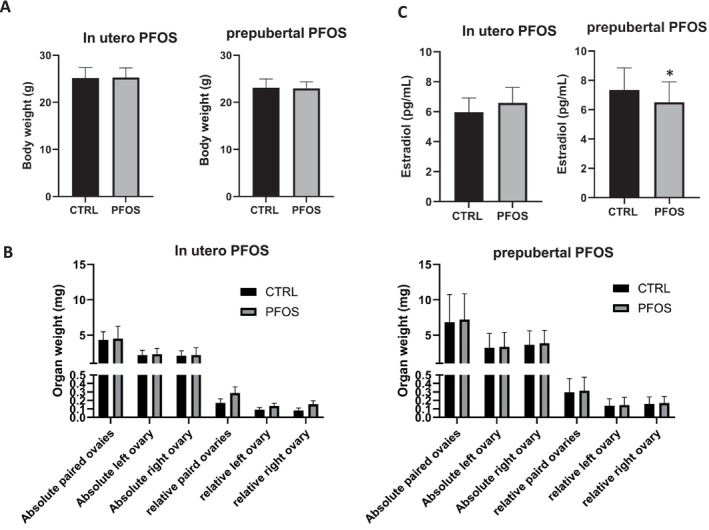
Effects of in utero and prepubertal exposure to PFOS on body weight (A), ovarian weights (B), and serum estradiol levels (C). The values represent the mean ± SD of 11–29 mice per group (prepubertal exposure: *n* = 22/control group, *n* = 29/PFOS group; in utero exposure: *n* = 17/control group, *n* = 11/PFOS group). *p < 0.05 when compared to the control.

**FIGURE 3:**
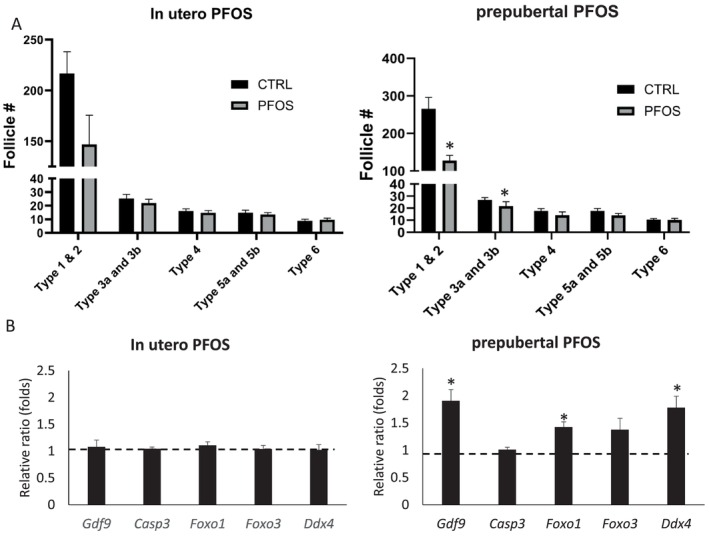
Follicle count (A) and transcript level expression of *Gdf9, Casp3, Foxo1, Foxo3, and Ddx4* in the ovaries of PND 28 offspring dosed prenatally or prepubertally with PFOS (B). (A) Follicles at PND 28 were counted and classified as described in Pedersen and Peters (1968). Types 1 and 2: Primordial, 3a and 3b: Primary, 4: Secondary (two layers), 5a and 5b: Tertial layers, and 6: Antral follicles. (B) Relative transcript levels of ovarian marker genes in PFOS‐exposed ovaries. The dotted line represents the baseline levels, assigned as one, representing no change compared with the control group. Left panels: In utero exposure; right panels: Prepubertal exposure. Values represent the mean ± SEM of 11–29 mice per group (prepubertal exposure: *n* = 22/control group, *n* = 29/PFOS group; in utero exposure: *n* = 16/control group, *n* = 11/PFOS group). *p < 0.05 when compared to the control group.

No statistically significant changes in body weight, absolute or relative ovarian weights, follicle counts, and serum estradiol levels were observed following in utero PFOS exposure (Figure 2A–C). No significant changes of the pup average body weights were observed between the control and the PFOS groups on PNDs 4, 7, 10, 14, and 21 (Figure S1). The total number of type 1 and 2 (primordial) follicles was lower in the PFOS‐exposed group. However, due to large individual differences that were observed, the changes were not statistically significant (Figure 3A). In addition, there were no observed changes in the transcript levels of *Gdf9, Casp3, Foxo1, Foxo3*, and *Ddx4* between the PFOS and control groups (Figure 3B).

### 3.2 Prepubertal Exposure

Prepubertal exposure to PFOS had no effect on body weight on PND 28 (Figure 2A) as well as on PNDs 7 through 21 (Figure S1). The paired absolute and relative weights of the ovaries in the PFOS‐exposed group were slightly higher than those of the control group; however, the difference was not statistically significant (Figure 2B). Serum estradiol levels in the PFOS‐exposed offspring were significantly lower than levels in the control group (Figure 2C). The number of types 1 and 2 (primordial follicles) and types 3a and 3b (primary) follicles observed per ovary was also significantly lower in the PFOS group relative to the controls (Figure 3A). Gene expression analysis indicated that transcript levels of *Foxo1* and *Gdf9* were both significantly higher upon PFOS exposure (*p* < 0.00005) (Figure 3B).

## 4 Discussion

Previously, work investigating PFOS and the effects on folliculogenesis was limited to examination of adult animals (Feng et al. 2015), potentially masking the effects of early exposure in developing animals. The present study examined the effects of in utero and prepubertal PFOS exposure on the first wave of follicle development in young mice.

The dose (0.1 mg/kg/day) of PFOS selected for this study was based on the published literature (Feng et al. 2015). The United States Environmental Protection Agency (EPA) established an interim updated health advisory level of PFOS in drinking water to be 0.02 ppt (0.02 pg/kg); the value was determined by evaluating data from mouse and rat studies (https://www.epa.gov/system/files/documents/2022‐06/drinking‐water‐ha‐pfas‐factsheet‐communities.pdf). Human occupational exposure to PFOS has been reported to range from levels of 0.33–4.22 pg/kg/day (Wee and Aris 2023). Thus, the concentration of PFOS used in the current study is expected to be higher (8.1 μg/kg/day) than normal human exposure levels when using the body surface area conversion described by Reagan‐Shaw et al. (2008). While PFOS exposure levels in this study exceed typical human exposures, the study nonetheless provides a useful starting point for evaluation of potential effects on folliculogenesis and the pathways/mechanisms that are targets for disruption.

At an exposure of 0.1 mg/kg, we found a significant difference in the number of primordial and primary follicles, serum estradiol levels and expression of the ovarian marker genes *Gdf9, Foxo1*, and *Ddx4* when PFOS was administered prepubertally. No changes, however, were noted upon in utero exposure. The impairment in folliculogenesis and reduction in estradiol levels upon postnatal exposure are consistent with previous reports (Feng et al. 2015; Wang et al. 2018). Feng et al. (2015) reported that exposure to PFOS affected the number of antral and preovulatory follicles at 0.1 mg/kg in adult mice, the same dose as used in the current study. However, our results in prepubertal mice revealed that primordial and primary (early) follicles were largely affected by PFOS exposure. The differences between the current study and the published literature may be related to the age of mice at exposure. The study by Feng et al. (2015) used adult mice, while we used pubertal mice (PND 28), which were undergoing the first wave of folliculogenesis. There are also less antral and preovulatory follicles in pubertal mice compared to adult mice, which makes evaluation of later stage follicles more of a challenge in the present study.

Expression of ovarian marker genes *Gdf9*, *Foxo1*, and *Ddx4* was also increased at the transcript levels in the PFOS‐exposed group relative to the control group. GDF9 is known to be important for follicle growth beyond the primary follicle stage and for oocyte maturation (Sanfins et al. 2018; Hreinsson et al. 2002; Yan et al. 2001). FOXO1 regulates proliferation and steroidogenesis in granulosa cells. An increase in FOXO1 expression has also been shown to accompany an increase in the expression of genes related to oxidative stress and apoptosis (Shen et al. 2012; Herndon et al. 2016). Granulosa cell apoptosis observed at low levels is part of the normal process for follicle development; however, higher levels of granulosa cell apoptosis have been shown to induce follicle atresia (follicle degradation) (Regan et al. 2018). DDX4 also has an important role in primordial germ cell specification and maintenance and is a recognized oocyte marker (Hickford et al. 2011). Deletion of *Ddx4* in fruit flies causes abnormal chromosome condensation of germline stem cells and checkpoint kinase 2 (Chk2)‐dependent oogenesis arrest (Durdevic and Ephrussi 2019; Pek and Kai 2011).

One potential hypothesis for a mechanism of PFOS disruption of primordial and primary follicle development is that the PFOS‐induced decrease in estradiol production impacts oocyte development via impairment of the granulosa cells. Additionally, increased transcription of *Foxo1* in the granulosa cells may impair ovarian follicle development through multiple processes including increased oxidation levels. Increased oxidation levels are associated with increased apoptosis levels in ovarian follicles (de Nigris et al. 2003). Our study also found that elevated levels of *Casp3*, suggesting prepubertal exposure to PFOS might induce apoptosis accompanied by increased oxidation levels in the mouse ovaries. In addition, the transcript levels of *Gdf9* and *Ddx4* also increased upon exposure to PFOS. Expression of both *Gdf9* and *Ddx4* is crucial for the proper development of oocytes, playing important roles in transcription and cell proliferation. The increased transcription levels of both genes by PFOS exposure suggest a protective role in normal follicle/oocyte development from the toxicity response. Following a general disruption of normal oocyte development and maturation signaling pathways, upregulated expression of both *Gdf9* and *Ddx4* genes may enhance follicle growth and oocyte maturation during recovery from injuries induced by drug toxicity (Ozcan et al. 2020). The current study is limited in that we only examined the first wave of folliculogenesis in prepubertal mice (PND 28) and did not investigate the second wave of folliculogenesis and more in adult mice. Examining the ovaries at a later stage during development is also crucial for understanding the full impact of PFOS exposure during the prepubertal period. The results of the current study of the first wave of folliculogenesis give rise to the following question: will changes in gene transcription, reduction in primordial follicle number and serum estradiol levels persist into the second or third wave of folliculogenesis or does the ovary have a recovery mechanism for the PFOS‐induced damage?

In utero exposure to PFOS did not induce detectable changes among the measured endpoints between control and exposed animals in the present study. Previously, transplacental exposure through prenatal dosing to PFOS has been reported to affect fetal weight in mice due to the impairment of placental transports of glucose and amino acids (Wan et al. 2020). However, the doses used were higher (3 mg/kg) than those used in the current study with administration occurring from GD 4.5 through GD 17.5. Although fetal weight was not examined in the present study, there were no changes in body weights of young pups in the PFOS‐dosed group compared to the control group. A prior study had also reported the no observed adverse effect levels (NOAEL) for PFOS as 0.1 mg/kg for in utero exposure (Dong et al. 2009).

## 5 Conclusions

The present study examined the potential effects of in utero and prepubertal exposure to 0.1 mg/kg PFOS on the first wave of folliculogenesis. Although there were no observed changes upon in utero exposure to PFOS in this study, several parameters including ovary weights, primordial and primary follicle number, serum estradiol levels, and the transcript levels of *Ddx4*, *Foxo1* and *Gdf9* were altered in the animals dosed prepubertally (PNDs 7–21) with PFOS. The preliminary findings suggest that prepubertal exposure to 0.1 mg/kg PFOS may affect follicle development by disrupting estradiol production. Increasing *Ddx4*, *Foxo1*, and *Gdf9* gene expression may protect follicle/oocyte development at the later stage of folliculogenesis. Further studies would be helpful, however, to confirm the current findings with a large number of animals and to fully examine the mechanisms underlying prepubertal exposure to PFOS on folliculogenesis. Moreover, this study conducted using animal models, which did not account for the differences in hormonal regulation on folliculogenesis between humans and animals (Recchia et al. 2021; Oduwole et al. 2021). Further studies exploring human‐based in vitro folliculogenesis models or new alternative methods (NAMs) could expand our understanding of PFOS‐induced mechanism of follicular toxicity. Additionally, the dose of PFOS that was used in this study was higher than the healthy advisory levels in humans, so additional studies including a dose equivalent to the advisory dose in humans also would be needed.

## Author Contributions

**Bounleut Phanavanh:** investigation (equal), writing – reviewing and editing (equal). **Jalina Moore:** investigation (equal), writing – reviewing and editing (equal). **Amy Inselman:** conceptualization (supporting), writing – reviewing and editing (equal). **Xiaoqing Li:** conceptualization (supporting), writing – reviewing and editing (equal). **Kyung Sung:** conceptualization (supporting), writing – reviewing and editing (equal). **Pei‐Hsuan Hung:** conceptualization (supporting), writing – reviewing and editing (equal). **Li You:** conceptualization (supporting), writing – reviewing and editing (equal). **Noriko Nakamura:** conceptualization, funding acquisition, investigation (lead), writing – original draft preparation, writing – reviewing and editing (equal).

## Funding

This study was supported in full by the Food and Drug Administration's Perinatal Health Center of Excellence (PHCE) funding program administered by the National Center for Toxicological Research.

## Disclosure

This presentation reflects the views of the authors and does not necessarily reflect those of the U.S. Food and Drug Administration. Any mention of commercial products is for clarification only and is not intended as approval, endorsement, or recommendation.

## Ethics Statement

All animal procedures were approved by the National Center for Toxicological Research Institutional Animal Care and Use Committee and followed the guidelines set forth by the National Research Council (2011) in the *Guide for the Care and Use of Laboratory Animals*.

## Conflicts of Interest

The authors declare no conflicts of interest.

## Supporting information


**Figure S1:** Average body weights of pups in utero and prepubertal exposures to PFOS at PNDs 4 through 21.
Values express as mean ± standard division. Body weights represent average of all pups per litter. In utero: *n* = 11–17 per group; prepubertal: *n* = 4–5 per group.

## Data Availability

The data that support the findings of this study are openly available in Figshare at https://doi.org/10.6084/m9.figshare.31955958.
