# Supplementary material for: Potential Effects of Prepubertal Exposure to Perfluorooctane Sulfonic Acid on the First Wave of Folliculogenesis in Young CD‐1 Mice
Source: Birth Defects Res. 2026 May 6;118:e70052. doi: 10.1002/bdr2.70052 (PMC13147321; doi:10.1002/bdr2.70052)
Supplement: Supplementary file 1 — Figure S1: Average body weights of pups in utero and prepubertal exposures to PFOS at PNDs 4 through 21. Values express as mean ± standard division. Body weights represent average of all pups per litter. In utero: n = 11–17 per group; prepubertal: n = 4–5 per group. [file BDR2-118-e70052-s001.pdf]

Supplemental Figure 1

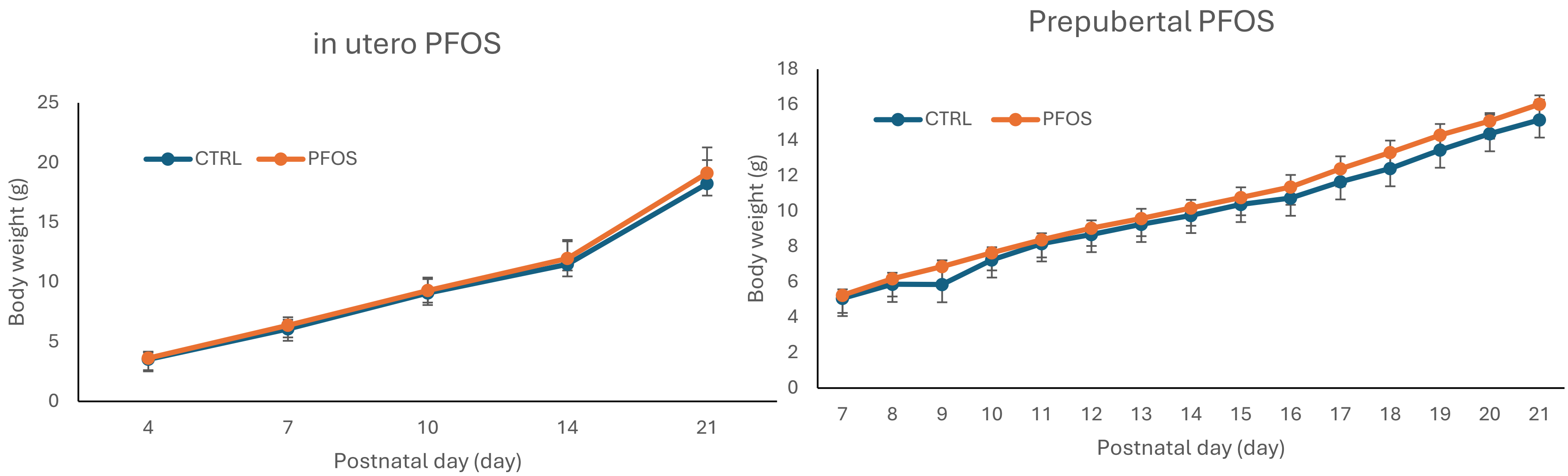

Supplemental Figure 1

Average body weights of pups in in utero and prepubertal exposures to PFOS at PNDs 4 through 21.

Values express as mean ± standard division. Body weights represent average of all pups per litter. In utero: n=11-17 per group; prepubertal: n=4-5 per group
